# A systematic review of tea pigments: Prevention of major diseases, protection of organs, and potential mechanisms and applications

**DOI:** 10.1002/fsn3.3666

**Published:** 2023-10-06

**Authors:** Xuan Zhao, Fei Yan, Xin‐Sheng Li, Dong Qu, Yue‐Ling Xu

**Affiliations:** ^1^ Qinba Black Tea Research Institute, Shaanxi University of Technology Hanzhong China; ^2^ Shaanxi Bio‐Resources Key Laboratory Hanzhong China; ^3^ Coordination and Innovation Center for Comprehensive Development of Qinba Biological Resources Hanzhong China; ^4^ College of Biological Science and Engineering Shaanxi University of Technology Hanzhong China

**Keywords:** functional mechanism, health benefits, industrial application, tea, tea pigment

## Abstract

With the growing awareness of a healthy life, tea pigments (TPGs) are in focus for their health benefits. TPGs not only provide specific color to tea liquor but also possess health benefits such as anti‐obesity, anti‐tumor, anti‐inflammatory, anti‐viral, anti‐oxidative, and bacteriostatic properties. Also, TPGs can benefit bone, liver, kidney, cardiovascular, gut microbiome, and sleep health. Based on previous reports, this review provides a brief introduction to the health benefits of TPGs, focusing on the prevention of human diseases and the protection of organs. Also, the latest research on the functional mechanism(s), practical application, and development strategies of TPGs is discussed.

## Abstract 2

Tea pigments (TPGs) prevent the metabolic syndrome and regulate intestinal microbes. Having special sensory qualities and health benefits, TPGs as functional ingredients have turned into a major research hotspot.

## 1 INTRODUCTION

**FIGURE 1:**
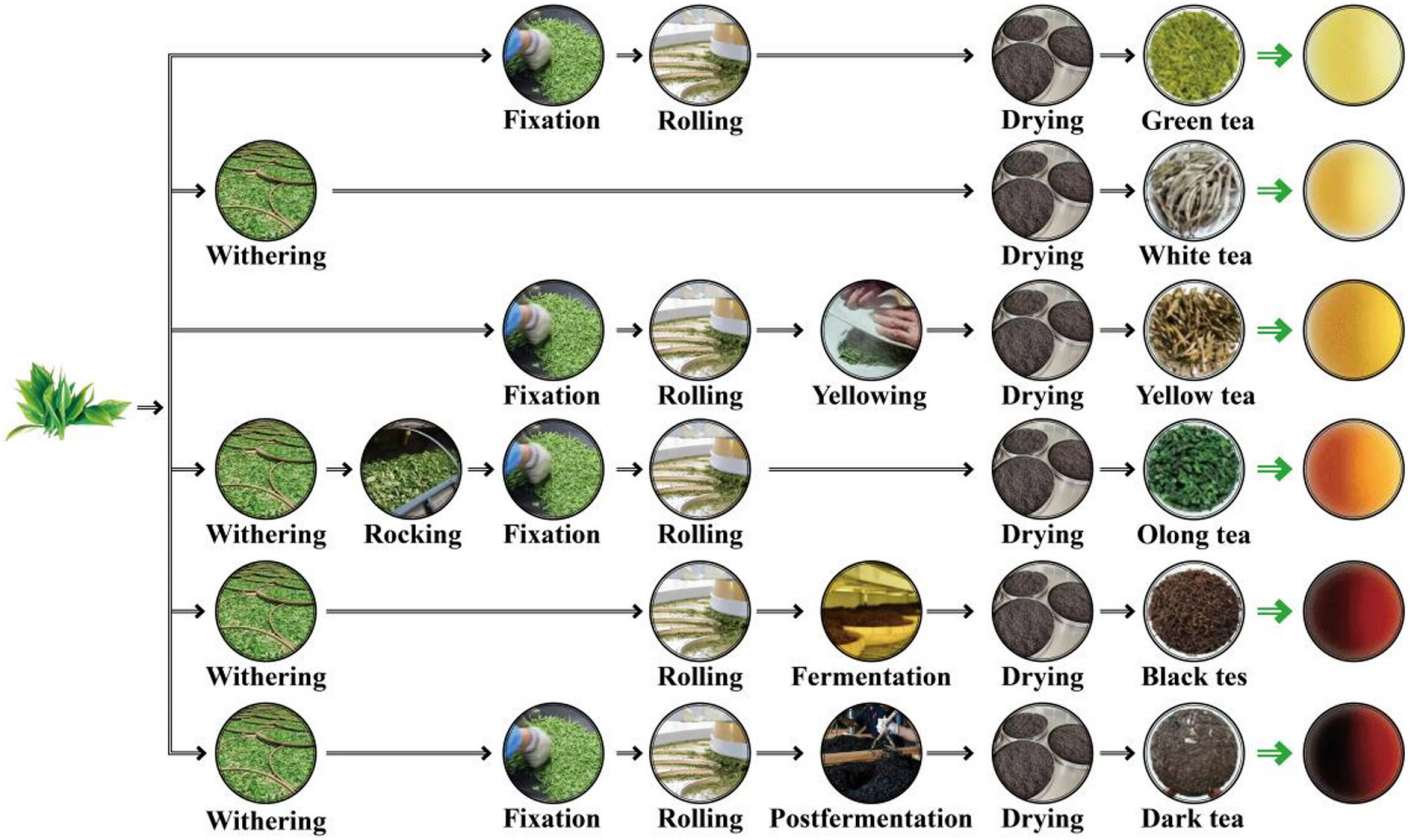
The general manufacturing processes of six types of tea (*Camellia sinensis*).

Tea (*Camellia sinensis* L.), consumed as a healthy beverage and widely recognized as a cash crop, has a planting history of more than 2100 years. Based on processing and degree of fermentation, tea can be divided into six categories: green tea, white tea, yellow tea, oolong tea, black tea, and dark tea (Figure 1). Black tea dominates the global tea market, contributing to 76%–78% of the total tea consumption. Tea is rich in functional components, such as tea polyphenols (TPs), catechins, tea pigments (TPGs), L‐theanine, tea polysaccharides, caffeine, and other bioactive substances (Chen, 2004; Gu et al., 2002; Wan et al., 2015), producing health benefits such as lowering blood lipid levels, blood pressure, and blood sugar, preventing atherosclerosis, protecting the liver and gallbladder, and having anti‐inflammatory and bacteriostasis properties (Jain et al., 2006; Li, 2004; Wang et al., 2013; Yuan & Dai, 2006).

TPGs are plant phenolic pigments that are produced from the action of polyphenol oxidase (PPO, EC 1.10.3.1), peroxidase (POD, EC 1.11.1.6), and other endogenous oxidases triggered by turgor loss due to water decrease during wilting of tea leaves. About 75% of TPs in tea leaves form o‐quinones via oxidative polymerization mediated by endogenous PPO and POD and then polymerize into theaflavins (TFs), thearubigins (TRs), and theabrownins (TBs). All of these determine the characteristics of fermented tea.

**FIGURE 2:**
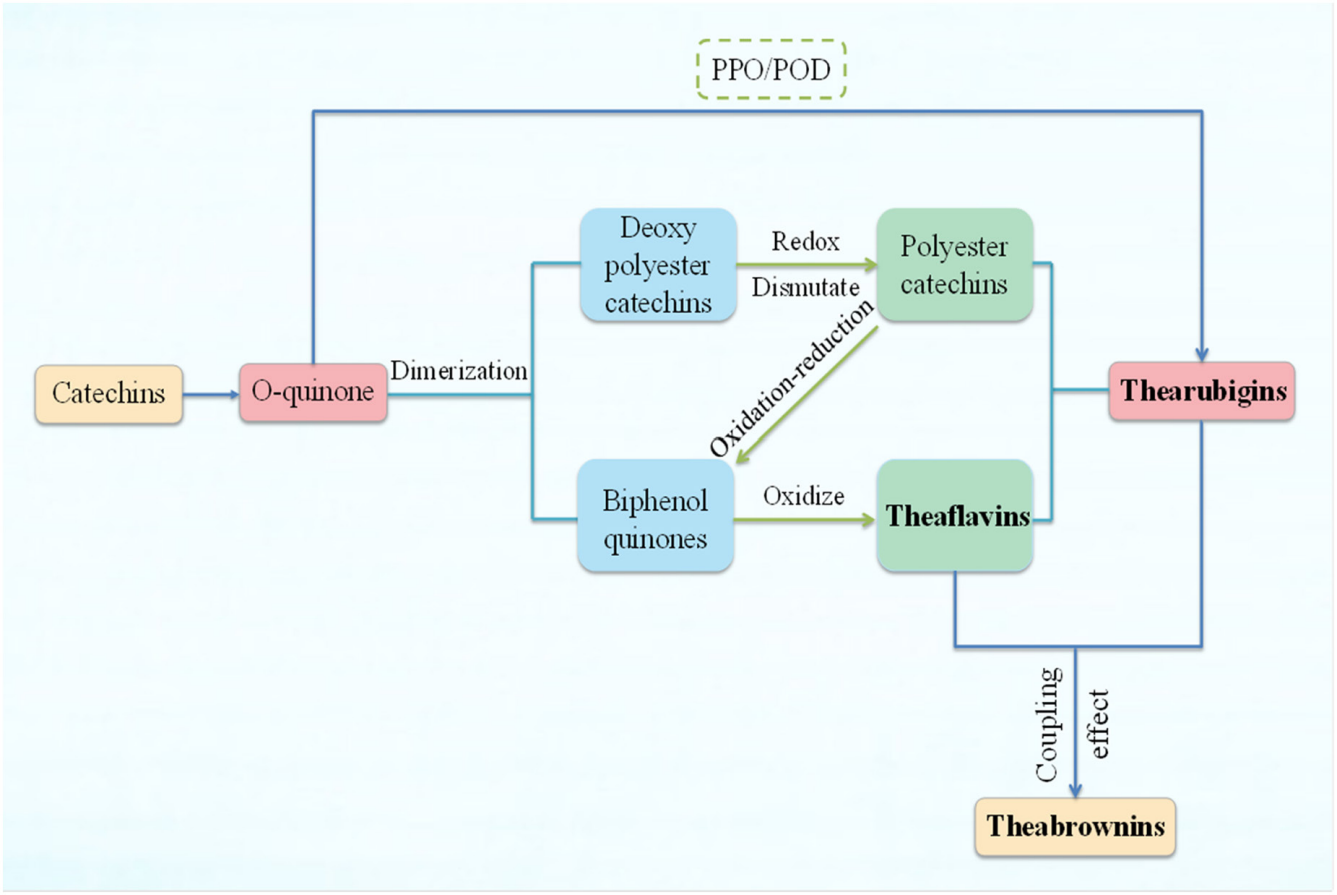
The pathway of catechins to form theaflavins, thearubigins, and theabrownins.

TF is a reddish‐orange pigment with a benzoketone chromophore, typical enzymatic oxidation products of catechins. It was first discovered by Roberts et al. (1957). The structure of TRs is still speculative, and Robert divides them into two broad categories based on the polarity of TRs, namely TRSI and TRSII. TFs and TRs are inextricably linked, and there are reports that TR comes from TFs during fermentation (Dong et al., 2018). TB is a kind of brown polymer with phenolic properties; the main components of polyphenols, caffeine, proteins, sugars, amino acids, and other oxidative polymerization products. Studies speculate that the formation route of TB is that endogenous enzymes such as catechol oxidase in tea first oxidize catechins and other phenolic substances to o‐quinone, then undergo oxidative polymerization of o‐quinones to form TFs and TRs, and then polymerize with other compounds to form TBs through coupling (Figure 2) (Zhang et al., 2017). In short, catechins are catalyzed by enzymes to form a variety of oxidation products, including TPGs. The enzymatic oxidation process of catechins is complex and diverse, which has important research significance; however, the oxidation pathway of catechins is single, the simultaneous analysis of multiple pathways is rarely involved, and the mechanism of the formation of TPGs needs to be further studied.

In general, only 0.6–1.6 kg of TPGs can be extracted from a ton of tea, and therefore TPGs are known as “gold in medicine” (Zhang et al., 2019). So far, four TF species have been discovered and identified (Mo et al., 2011), of which TF, theaflavin‐3‐gallate (TF‐3‐G), theaflavin‐3′‐gallate (TF‐3′‐G), and theaflavins‐3,3′‐gallate (TFDG) are the major ones (Liu, Zhang, et al., 2021). Higher content of TFs improves tea color, seen as a “golden circle” on the glass rim (Margaret et al., 1981). TRs are the main contributor to the color of black tea water, accounting for 9%–19% of the dry weight of black tea (Chen, 2018a; Hu et al., 2014; Wang, 2007). TFs and TRs are oxidized and polymerized to form water‐soluble, non‐dialyzable, highly polymerized brown substances (Cheng & Ruan, 1983; He et al., 2019), namely TBs. Selected data from the literature are compiled in Table 1.

**TABLE 1:** The contents of theaflavin (TF), thearubigin (TR), and theabrownin (TB) (%).

Sample	Theaflavin	Thearubigin	Theabrownins	References
Darjeeling teas	0.28–0.56	3.625	13.093	Engelhardt (2013)
				Nishimura et al. (2007)
				Owuor et al. (1986)
				Khanum et al. (2017)
Assam teas	0.96–1.91	7.257	12.247	Engelhardt (2013), Davis et al. (1997), Owuor et al. (1986)
				Khanum et al. (2017)
Sri Lankan teas	0.61–1.15	8.40	5.54	Engelhardt (2013)
				Yi et al. (2017)
African teas	1.66–2.30	9.80	5.63	Engelhardt (2013)
				Yi et al. (2017)
Chinese teas	0.44–0.89	8.21	7.34	Engelhardt (2013)
				Hu, Li, et al. (2021)
Earl Gray	0.12–0.26	—	—	Nishimura et al. (2007)
Ceylon	0.20–0.25	—	—	Nishimura et al. (2007)

At present, experimental and clinical studies have shown that the healthcare benefits of tea are mainly composed of tea polyphenols and derivatives, such as TF, TR, and TB, tea polysaccharides, amino acids, and other functional components. The extraction of TPGs and the application of TPGs in food field and other fields have been the subject of a lot of research and practice in the international arena. With the changes and needs of the food and drug market, TPG has the characteristics of a wide source, a simple preparation process, low cost, good safety, and stability, and its production and industrialization have become an inevitable trend in the development of the industry (Hu et al., 2014; Liu, Bruijn, et al., 2021; Liu, Zhang, et al., 2021; Wu et al., 2019). China, with its abundant tea resources, is the world's largest exporter of tea. Currently, TPs, theanine, TPGs, and other tea extracts in international circulation are mostly exported from China to be processed into functional foods. However, there are only a few functional tea medicines and foods on the domestic market in China. Scientifically utilizing China's tea resources, identifying biologically active tea ingredients, and producing reliable tea series as health foods, medicines, and beauty products can be promising prospects. Therefore, on the basis of reviewing the past literature, this paper comprehensively reviews the health benefits of several TPGs, in order to provide development ideas for the research and development and application of TPGs.

**FIGURE 3:**
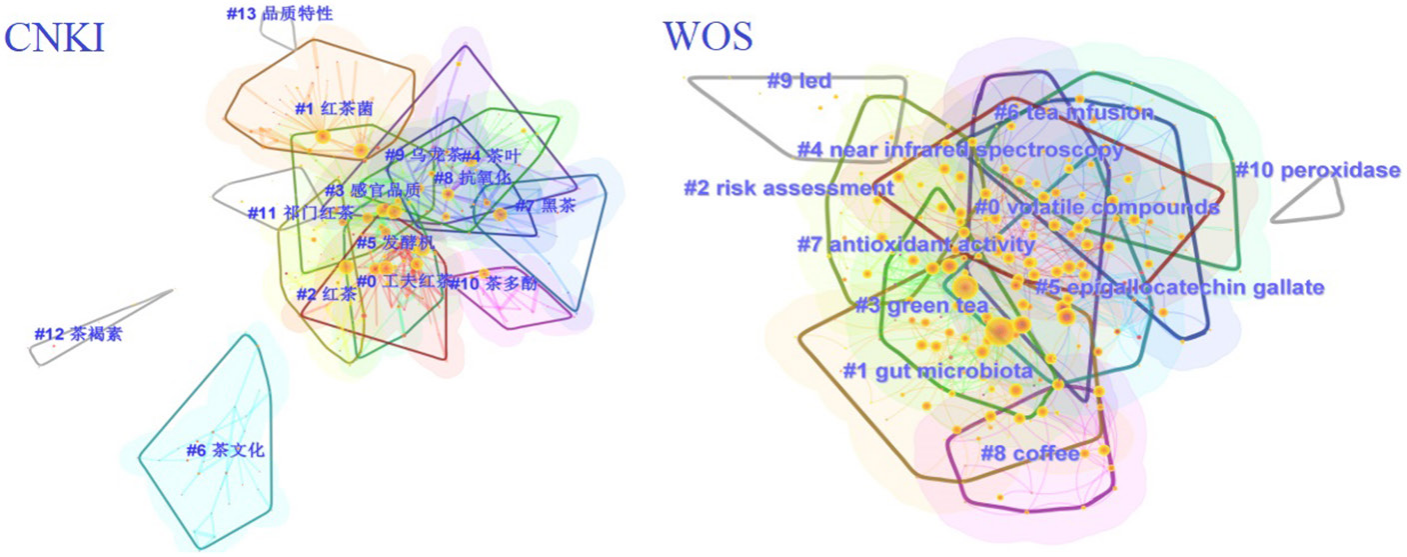
Keywords clustering map of tea pigments research in English and Chinese literature.

Herein, this paper conducted a CiteSpace visual analysis of more than 3000 pieces of past literature on fermented tea since 2012. Using 1412 CNKI (China National Knowledge Infrastructure) documents and 2355 core set documents of WOS (Web of Science) as data sources, a scientific knowledge map of TPGs research was constructed; the visual analysis was carried out mainly in terms of keyword clustering. A higher keyword frequency indicates a research hotspot in this field. The keywords clustering map of the fermented tea is based on data obtained from the CNKI and WOS databases (Figure 3). Different colors represent different clusters. The higher the number of keywords, the higher the cluster number, indicating general concerns in TPG research. The two databases were aggregated into 13 and 9 categories, respectively, based on keywords such as gut microbiota, antioxidant activity, TPs, epigallocatechin gallate, near‐infrared spectroscopy, quality components, biological activities, and experimental methods (Figure 3). This analysis indicates that the research on TPGs mainly focused on disease prevention, mechanism research, preparative separation, and quality components. Based on some hotspots and development trends, this study reviews the biological activities of TPGs, focusing on their applications in the fields of health food, medicine, and cosmetics. This review provides a reference for the comprehensive application of TPGs.

## 2 HEALTH BENEFITS OF TPGS

**FIGURE 4:**
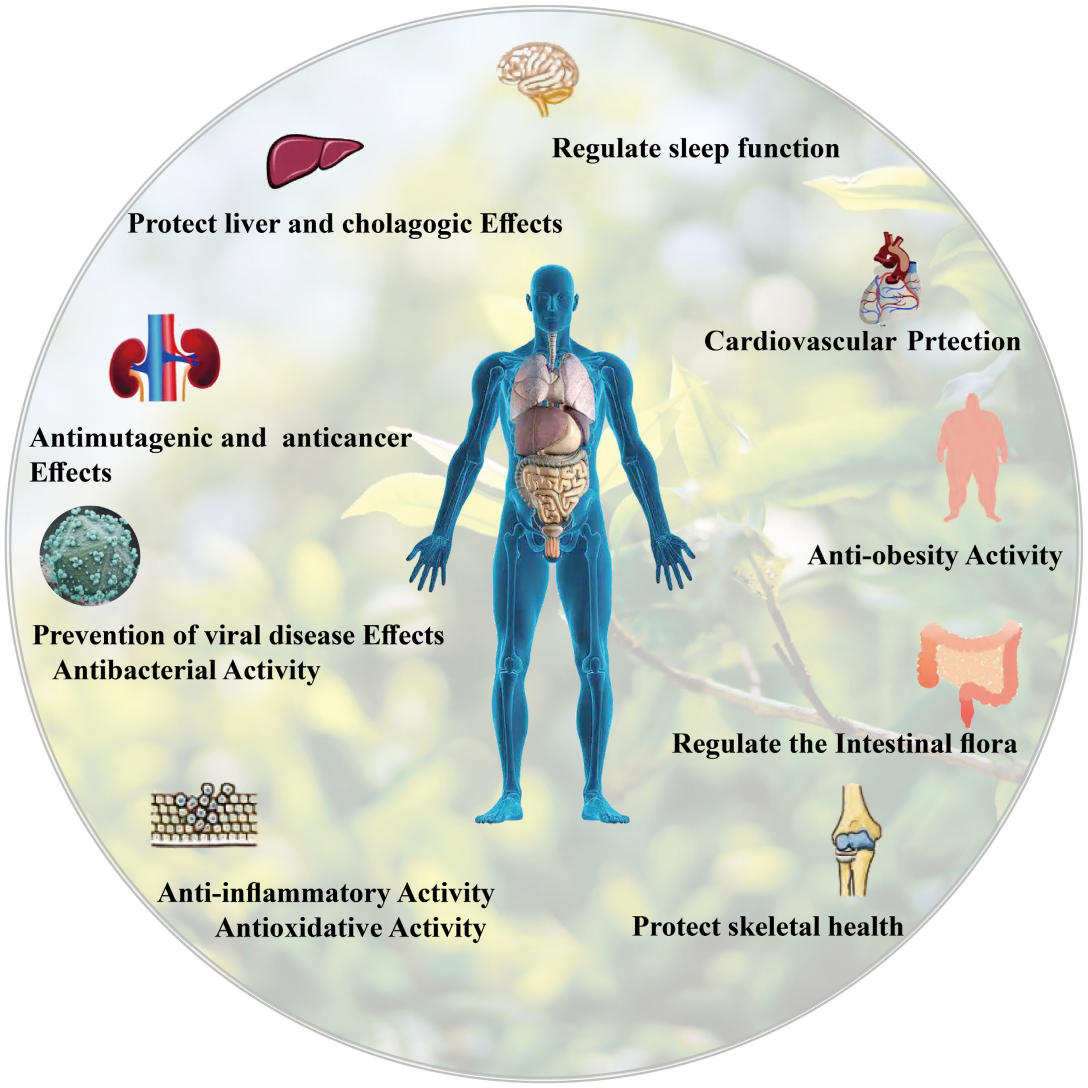
The health benefits of TPGs.

Fermented teas have more health benefits than regular teas (Figure 4); TPGs are the characteristics of a fermented tea. TFs, TRs, and TBs have been investigated for their potential health benefits in fermented tea. However, it is not yet fully clear how TPGs in fermented teas confer specific biological activities. With good water and fat solubility and complex structure and composition, It can reach the colon in the form of polymers, which are then decomposed by the intestinal microflora to produce phenolic acids (PAs) or other small‐molecule metabolites (Jiang, 2018). Therefore, TPGs improve intestinal microbial communities, which, in turn, provide health benefits to the host.

### 2.1 Prevention of major diseases in humans

#### 2.1.1 Anti‐obesity activity

Weight loss is gaining significant attention from health professionals to counter the increasing problem of overweight and obesity. Understanding the lipid‐lowering effects of fermented tea, its main components, and its underlying mechanisms of action are the major hotspots of current tea research. A study in high‐fat diet (HFD) rats showed that TPGs (TFs, TRs, and TBs) administered by gavage reduced the liver cholesterol level, lowered blood lipids, and in turn alleviated the effects of obesity. Also, TRs promoted the excretion of acidic steroids, lowering the content of liver lipids (Kianoosh et al., 2006). Chen et al. (2011)) and Wang and Gong (2012) found that TBs from Pu'er tea significantly reduced the cholesterol level in HFD rats and inhibited body weight growth and hepatic steatosis. TBs bind cholate, improving cholesterol and lipid absorption, which promotes cholesterol conversion into bile acids, thereby producing the lipid‐lowering function (Gong et al., 2020).

Ji‐Eun et al. (2009) and Kobayashi et al. (2009) performed in vitro assays with triglycerides and mixtures of triglycerides, phosphatidylcholine, and sodium taurocholate as substrates, and found that TFs effectively inhibited pancreatic lipase; the order of inhibitory effect was TF‐3′‐G > TF‐3‐3′‐G > TF‐3‐G. The pancreatic lipase inhibiting efficiency of TF‐3,3′‐G was stronger than that of epigallocatechin gallate (EGCG) and epicatechin gallate (ECG) alone or in combination. Du Yatao et al. (2005) showed that TFs effectively inhibited the activity of fatty acid synthase (FAS) and reduced the decomposition of fat in the intestine, thereby reducing fat absorption in the body. Li et al. (2018), using the p‐nitrophenol method, showed that black tea TBs had a significant inhibitory effect on lipase and can regulate the key enzymes accelerating lipid metabolism, which may reduce the damage caused by high‐sugar diets to pancreatic islets (Zhao et al., 2019). Current studies suggest that TBs intervention changes lipid metabolism‐related gene expressions and pathways in mature adipocytes (Liu, Feng, et al., 2018). Zhang et al. (2018), Zhang, Miao, et al. (2022) believe that TBs exert lipid‐lowering and weight‐loss effects by upregulating the *Cyp7a1* gene (the rate‐limiting enzymes in the classical pathway of bile acid anabolism), which accelerates the catabolism of dietary cholesterol, improving energy consumption, and leading to weight loss. Meanwhile, downregulation of *Angptl‐4* (stimulates sugar uptake in the liver, improves insulin resistance, and promotes gluconeogenesis) and *CYP4A8* (lipid peroxidase) genes was shown to reduce serum FPG (fasting plasma glucose) and total cholesterol (TG) levels in rats.

As discussed above, TFs, TRs, and TBs all have good lipid‐lowering and weight‐loss effects. However, the strategies to maximize these effects need further research to improve our understanding of the underlying mechanisms.

#### 2.1.2 Antimutagenic and anticancer effects

When a certain somatic cell loses regulatory control, it continues to proliferate, eventually forming a malignant tumor. In the study of human prostate tumor cells, Sakamoto (2000)) found that TR with genistein (trihydroxyisoflavone) inhibited the growth of human prostate tumor cells in a synergistic manner but failed to produce the same effect alone. Dhawan et al. (2002) reported that both TFs and TRs blocked heterocyclic amines‐induced rupture of human lymphocytes without affecting normal human lymphocytes. Liang et al. (1999) showed that TRs inhibited the autophosphorylation of epidermal growth factor receptors. Halder et al. (2006) found that TRs have a good anti‐teratogenic effect against human lymphocytes. In addition, TRs were shown to block the activity of cellular enzymes and inhibit the formation of benzopyrene‐induced adducts, which is a cancer chemoprevention effect (Krishnan & Maru, 2005).

In addition, TPGs inhibited FAS activity both at the protein and mRNA levels in HepG2 hepatoma cells, thereby controlling liver metastases in experimental mice (Chiang et al., 2006; Yang et al., 2012). Catterall et al., (Catterall et al., 2003) found through animal experiments that the TFs and glutathione combination can activate intermediate aflatoxin B_1_8,9‐oxide, enhancing AFB1 activity before a series of hepatic S9 activation reactions. Tu and Tang (2004) studied the effects of TFs, TFDG, and theaflavin‐3‐gallate (TF2B) on gastric carcinoma (MKN‐288), hepatoma (BEL‐7402), and leukemia (LH‐60) cells and found significant inhibitory effects of TF2B on all three cells, while the inhibitory effect of TFDG on MKN‐288 and BEL‐7402 cells was higher than that of TFs.

Many studies have shown that TFs and TRs can significantly control the growth and metastasis of tumor cells, but most of these studies used in vitro experiments. At present, there are still many challenges in the clinical application of TF, TR, and TB as anticancer inhibitors, such as the fact that the anti‐cancer mechanism of teapigments has not been perfected, the stability of TFs and TRs is poor, and the potential toxicity is not clear. In short, TPGs as an anti‐cancer drug needs more research to prove.

#### 2.1.3 Anti‐inflammatory activity

Inflammation is the first biological immune response to infection, injury, or stimulation. Tea exerts anti‐inflammatory actions by regulating immune cells and reducing the expression of inflammatory cytokines. Polyphenols (catechins), TPGs, and tea polysaccharides are the main anti‐inflammatory components in tea.

A study in mice showed that the volatile components from green, yellow, and Pu'er teas produced significantly stronger anti‐inflammatory effects than those from white, oolong, and black teas (Han, 2018). Zijuan tea extract significantly reduced the secretion of inflammatory factors in mouse macrophages and RAW264.7 cells, thereby reducing the production of inflammatory mediators (Li et al., 2020). Fuzhuan tea extract blocked the inflammatory pathway, improved the intestinal flora, and reduced the expression of inflammatory factors, alleviating dextran sulfate sodium‐induced ulcerative colitis in mice (Huang et al., 2021). TRs significantly reduced diarrhea and colorectal injury in trinitrobenzene sulfonic acid‐induced colitis mice by reducing the osmotic injury to neutrophils, decreasing lipid peroxidation, inhibiting serine protease, and releasing superoxide anion radicals (Swapna et al., 2003). Furthermore, TFs protected mouse chondrocytes from apoptosis and senescence by modulating the Keap1/Nrf2/HO‐1 axis (Hegarty et al., 2000) and modulating the tert‐butyl hydroperoxide‐induced anabolic and catabolic imbalance of primary chondrocytes in osteoarthritis mice (Yoshiomi et al., 2011).

Aged Pu'er tea can inhibit the intestinal oxidative stress‐mediated inflammatory signaling pathway (TLR4/MyD88/ROS/p38MAPK/NF‐κBp65) by upregulating the intestinal tight junction proteins (MUC‐2, ZO‐1, occluding) and M2 polarization of macrophages, which in turn improves intestinal immune barrier and reduces intestinal inflammation (Hu, Chen, et al., 2021). Hou et al. (2021) found that TFDG effectively inhibits the secretion of pro‐inflammatory cytokines but promotes the secretion of anti‐inflammatory cytokines. TFs were shown to prevent inflammation by inhibiting the expression of cyclooxygenase‐2 (COX‐2), downregulating pro‐inflammatory cytokines (TNF‐α), and blocking the expressions of iNOS (inducible nitric oxide synthase) and NF‐κB (Liu & Li, 2019). Also, TFs can reduce the expression of interleukin‐6 (IL‐6) and other key factors to block the NF‐κβ signaling pathway, inhibiting the degradation of intracellular IκBα, and blocking the nuclear translocation of RelA (NF‐κβ‐p65), thereby inhibiting inflammation (Fu et al., 2018; Wu et al., 2017). TFs attenuated the inflammatory response and cerebral hemorrhagic brain injury in the chronic MPTP/probenecid model of Parkinson's disease by inhibiting NF‐κB and other signaling pathways (Anandhan et al., 2013).

The anti‐inflammatory action of TPGs mainly involves the inhibition of inflammatory factors, regulation of inflammatory signaling pathways, and promotion of anti‐inflammatory cytokines. However, limiting ourselves to in vitro and animal tests without enough clinical studies, the underlying anti‐inflammatory molecular mechanisms of TPGs are still largely unclear and require more effort.

#### 2.1.4 Antiviral activity

Measles, rubella, mumps, chickenpox, coronavirus pneumonia, AIDS, viral hepatitis, and influenza are viral diseases. Most viral diseases are contagious and lack a good prognosis.

An in vitro study showed that TRs can prevent leukemia by controlling the first phase of cell division, regulating the expression of intracellular protease genes, and inhibiting the proliferation of chronic myelogenous leukocytes (Li et al., 2016). Another study showed that both TRs and TFs can inhibit the growth and synthesis of myeloid leukemia and chronic myeloid leukemia cells (Das et al., 2002).

In Vero and A549 human non‐small cell lung carcinoma cells, TFs, especially TFDG, were shown to inhibit genital ulcers caused by simplex type 1 and type 2 herpes viruses at pH <5.7 but >4.5 (Isaacs & Xu, 2013; Oliveira et al., 2015). Meanwhile, TFs can inhibit the neuraminidase activity of the highly pathogenic avian influenza (H5N1) virus by binding to its hemagglutinin HA2 subunit (Yang et al., 2014). Out of 720 natural compounds, only TFDG effectively inhibited chymotrypsin (3CL Pro) to prevent/treat severe acute respiratory syndrome (Severe Acute Respiratory Syndrome, SARS) (Chen, Coney, et al., 2005).

Cao et al., (Cao et al., 1999) showed that TRs can resist HIV infection by having a different mechanism for inhibiting the reverse transcriptase and DNA and RNA polymerase activities of HIV‐1. Similarly, Yang Jie et al. also showed that a high concentration of TFs inhibited the reverse transcriptase activity of HIV, and could be used as a second‐generation microbicide to prevent the spread of HIV (Yang, 2010). In addition, TRs were shown to have an inhibitory effect on toxins. Satoh et al. (2001, 2002) showed that TR n‐butanol solution inhibited botulinum toxin type A in a mouse model and therefore can be used to prevent tetanus. Chen et al. (2023) found that TF potently inhibited ASFV replication at non‐cytotoxic concentrations ex vivo in primary porcine alveolar macrophages (PAMs).

#### 2.1.5 Protection of skeletal health

The adult human skeletal system consists of 206 bones and more than 200 joints, accounting for about 20% of the body weight. The health of the skeletal system is the foundation of human health. Tea is good for bone health and can prevent osteoporosis and hip fractures (Wu et al., 2002) by increasing bone mineral density (BMD) (Chen et al., 2003). Some studies showed that tea consumption reduced the risk of osteoporotic fractures in elderly women and improved BMD in postmenopausal women (Wang et al., 2019). TFDG was shown to suppress osteoclastogenesis and osteoclast bone resorption by inhibiting the ERK pathway (Wu et al., 2017). Das et al. (2005) found that black tea extract alleviated osteoporosis in ovariectomized rats by increasing the levels of serum estradiol. Consistently, Liang et al. (2018) also showed that TRs treatment significantly improved cortical bone thickness and bone resorption biomarker levels in ovariectomized rats without affecting body weight. Furthermore, an in vitro study showed that TRs inhibited osteoclastogenesis and reduced the expression of related genes. Overall, the above studies suggest that TRs supplementation can alleviate the problem of osteoporosis in menopausal females.

Hou Zhenyang et al. showed that TFDG effectively inhibited the expression of pro‐inflammatory cytokines, induced M1 to M2 polarization in macrophages, promoted bone formation, and thereby reduced bone loss in collagen‐induced arthritis (CIA) mice; TFDG can be an ideal drug for the prevention and treatment of rheumatoid arthritis (Hou, 2021). Studies showed that TFs can reduce hormone‐induced lipid dysmetabolism to prevent avascular necrosis of the femoral head (Chen, Coney, et al., 2005, Chen, Ho, et al., 2005). Also, TFs can promote the expression of Cbfa1/Runx2, which are important regulators of osteocalcin (OCN), osteopontin, bone sialoprotein, type I collagen, and other osteogenic genes, promoting osteoblast differentiation (Li, 2018). It is proposed that TFs induce osteogenic differentiation of bone marrow mesenchymal stem cells (BMSCs) by promoting the expression of *Cbfa1/Runx2* OCN and alkaline phosphate (ALP), inhibiting the expression of the adipogenic gene, and promoting the expression of the osteogenic gene *Pcp4* (Shang et al., 2010).

With global aging, osteoporosis has become a serious public health concern. TPGs, having the properties of reducing pro‐inflammatory inflammatory factors and inhibiting bone loss, can be helpful in the management of osteoporosis.

### 2.2 Protection of organs

#### 2.2.1 Liver protection and cholagogic effect

The liver, gallbladder, pancreas, and kidneys are the important organs of the digestive and urinary systems; the dysfunction of these organs can lead to various diseases.

Chen et al. (2015) found that treatment with Ninghong black tea lowered the serum levels of total cholesterol, low‐density lipoprotein‐C, liver malondialdehyde (MDA), and liver index in hyperlipidemic mice. Moreover, the black tea extract also alleviated the hepatic lesions, which are characteristics of non‐alcoholic fatty liver (Shen et al., 2020). Mechanistically, black tea promoted the expression of PPARα and microsomal triglyceride transfer protein in the non‐alcoholic fatty liver of rats, thereby promoting fatty acid β oxidation and the synthesis of very low‐density lipoprotein. In HFD rats, TBs significantly reduced blood lipid levels by regulating the gene expression of lipid metabolism‐related enzymes (Fan et al., 2017).

The lipid‐lowering effect of TPGs in hepatocytes was dose‐related, i.e., the lipid‐lowering effect of TFs was reduced after a high dose (Zhang et al., 2014). Kobayashi et al., 2009 showed that black tea polyphenols inhibited pancreatic lipase activity; TFs monomer TFDG had a better inhibitory effect than EGCG, ECG, or an EGCG–ECG mixture (Kobayashi et al., 2009). Li et al. (2021) showed that TFs can improve insulin secretion in damaged islet cells. TRs were shown to regulate the diversity and abundance of gut microbes, which improved insulin resistance and the adverse effects of diabetes in hyperglycemic rats (Yue et al., 2019). TFs reduced the uric acid content in hyperuricemia mice in a dose‐dependent manner (Liu et al., 2020). Takashima et al. (2021) showed that TFs inhibited bile acid transporters and reduced the plasma level of cholesterol. TRs were shown to attenuate hypercholesterolemia by modulating intestinal microflora and bile acid metabolism in mice and humans (Huang et al., 2019).

Although TPGs were shown to alleviate liver injury, improve the adverse effects of diabetes, and lower uric acid, the underlying mechanism is not yet clear. Also, most of these effects have not been evaluated in clinical settings, and therefore require careful assessment.

#### 2.2.2 Cardiovascular protection

According to a 2019 report from the World Health Organization, cardiovascular diseases (CVDs) have been the leading cause of global mortality and disability in the past 30 years (Giles et al., 2009). A large number of intervention studies suggest that moderate tea drinking can improve cardiovascular health. Results indicated that (Zhang, Miao, et al., 2022, Zhang, Qu, et al., 2022) TF‐1 is a powerful inhibitor of platelet activation and thrombosis formation in C57BL/6 mice and could be developed as a novel food‐based inhibitor of thrombotic disorders. TFs reduced high‐fat‐induced oxidative stress injury of vascular endothelial cells by activating the Nrf2/HO‐1 pathway, and thereby slowed the pathological progress of atherosclerosis in HFD mice (Zeng, 2021). Chen et al. (2015) showed that black tea, in a dose‐dependent manner, increased the activities of serum lipoprotein lipase, hepatic triglyceride lipase, and total lipase in HFD mice. In addition, studies showed that black tea has antihypertensive effects and improves endothelial dysfunction in rats (Alkerwi et al., 2015; Tong et al., 2014). TFs were shown to reduce the levels of reactive oxygen species and MDA, protect vascular endothelial cells, and improve atherosclerosis (Zeng et al., 2021).

In the above studies, animal in vitro models were often used to examine the cardiovascular protective effect of TPGs, but no pharmacokinetic studies were performed in humans. Although the efficacy of TPGs in preventing cardiovascular and cerebrovascular diseases has been fully proven, their exact mechanism of action remains to be elucidated. Therefore, more research is needed to explain the mechanism of action of TFs, TRs, and TBs in the prevention and treatment of CVDs and to guide the development of functional foods and drugs based on TPGs.

### 2.3 The mechanism of TPGs

#### 2.3.1 Effect on intestinal flora

Intestinal microflora plays an important role in human health and has a broad‐spectrum substrate degradation ability. Disturbances in gut microflora can lead to various diseases, including obesity (Gill et al., 2006).

Li et al. (2023) found that TFs effectively improve behavioral impairment via the microbiota–gut–brain axis and upregulate brain neurotrophic factors. Jiang et al., 2018 used high‐throughput sequencing and bioinformatics analysis to find that intragastrically administered TFs, TRs, and TBs improved the species abundance, diversity, and structure of intestinal flora in HFD rats (Jiang et al., 2018). Moreover, the relative abundance of Firmicutes decreased and that of Bacteroidetes increased at the phylum level, and at the genus level, the relative abundance of Ruminococcaceae decreased and that of Lactobacillus, Akkermansia, and Lachnospiraceae increased to varying degrees. Yue et al., 2016 treated HFD mice with liubao tea for 8 months and then measured changes in bacterial gene composition in the feces. They found that TBs improved the structure of mice's intestinal flora, altering the quantity of Bacteroides and Sclerobacteria (Yue et al., 2016). Cai et al. (2021) showed that TFs inhibited lipid synthesis and accumulation in the liver in mice. TFs were also shown to regulate glucose and lipid metabolism in mice, inhibit the function of adipocytes and improve the abundance of healthy intestinal microflora, reducing obesity (Sirotkin & Kolesarova, 2021). Liu, Bruijn, et al. (2021) showed that TF TFDG promoted the relative abundance of Faecalbacterium, Parabacteroides, Bifidobacterium, and Bacteroidetes, and inhibited the proliferation of Prevotella and Fusobacterium in human intestinal flora. Studies showed that TFs can alleviate diabetes and dyslipidemia by modulating the composition of gut microbes (Kashif et al., 2021; Ma et al., 2022). Liao, 2021 performed in vitro anaerobic fermentation experiments and found that black tea, a new type of prebiotic, can regulate human intestinal flora. Also, the authors suggested that the mechanism of action of black tea is different from the common prebiotic fructooligosaccharide (Liao, 2021). Fructooligosaccharides mainly promote the production of acetic acid and increase the abundance of bifidobacterium, while black tea promotes the production of acetic, propionic, and n‐butyric acids, in turn increasing the growth of short‐chain fatty acids‐producing Bacteroides and Roseburia.

Recent studies suggest that most TRs are not digested in the upper gastrointestinal tract and reach the colon, where these TPGs are decomposed by the gut microbiota into phenolic acids or other small‐molecule metabolites (Liu, Bruins, et al., 2018; Liu, Feng, et al., 2018). There seems to be two‐way communication between TRs and gut microbiota, affecting host health (Zhu et al., 2021).

#### 2.3.2 Regulation of sleep function

Sleep is important for physical and mental health. However, in the current lifestyle, mental stress and a lack of exercise are causing circadian rhythm disorders (CRD, also known as sleep disorders), which negatively impact the normal work–life balance. Epidemiological investigations suggest that worldwide, more than 10% of people suffer from insomnia and more than 25% suffer from transient or occasional insomnia (Dew et al., 2003; Vgontzas et al., 2013). Various sleep disorders may exacerbate infectious and immune diseases. Therefore, people have now begun to pay serious attention to sleep health.

TPGs contain a large number of phenyl groups, and catechin has at least one benzene ring (Sato et al., 2012). Studies suggest that TPGs can combine with tea caffeine to reduce its absorption, which can alleviate the problem of poor sleep from caffeine consumption (Song et al., 2013). Mazzotti et al. (2011) demonstrated that the stimulating effect of caffeine was inhibited when complexed with TPGs.

Also, TPGs can modulate the brain‐gut axis via the immune system and gut microbes, regulating sleep health. Guo et al. (2019)) showed that TPS, theanine, and TFs regulated the expression of liver clock genes in mice. Furthermore, Hu, Chen, et al., 2021; Hu, Li, et al. (2021) confirmed that Pu'er tea increased bile acids to reduce CRD‐induced obesity in mice. Bile acids can remodel the gut microbiota, thereby reducing intestinal inflammation and CRD‐induced oxidative stress. It is suggested that PRT can mediate enterohepatic circulation to alleviate CRD injury by improving bile acids.

For now, TPGs are believed to improve sleep health via the brain–gut axis or by creating TPG–caffeine complexes to reduce caffeine absorption. In the future, TPGs' mechanisms of action can be explored from the aspects of monoamine neurotransmitters, inflammatory factors, and neurotrophic factors. Animal models can be used to develop sleep‐regulating tea foods.

#### 2.3.3 Antibacterial activity

The ancient medical books of the Tang and Song dynasties mention that tea could sterilize and inhibit bacteria (Chen, 2018a, 2018b). With the advancement of modern scientific research, the bacteriostatic effect of tea has been gradually improved. It is now widely used in drug research and development, beauty cosmetics, and as a food preservative.

TFs possess strong antibacterial abilities. TFs were shown to inhibit the growth and acid‐producing ability of the main cariogenic bacteria in the oral cavity (Jin et al., 2011). At a low concentration of 0.6–1.7 μmol/L, TFs inhibited 50% of α‐amylase activity and showed a strong inhibitory effect on glucosyltransferase of *Streptococcus mutans* (Lin & Yao, 2011; Zhang & Kashket, 1998). TFs have a strong affinity for bacterial α‐amylase. Upon binding to TFs, α‐amylase loses its activity, which inhibits bacterial growth. A study showed that sub‐inhibitory concentrations of TFs significantly reduced the level of the cariogenic virulence factor *S. mutans* (Kong, 2022). TFs also have bacteriostatic effects against a variety of Gram‐positive and Gram‐negative bacteria, including multiple drug resistance (MDR) strains (Diao et al., 2017). Nosocomial infections, such as *Stenotrophomonas maltophilia* and *Acinetobacter baumannii*, are resistant to most antibiotics. Interestingly, TFs have an obvious bacteriostatic effect on these pathogens, and the effect is more significant when used with epicatechin (EC) (Mao, 2021).

Compared with green tea, black tea has a more pronounced antibacterial effect. The minimum inhibitory concentrations of green and black teas against *S. mutans* were 150 and 50 mg/mL, respectively (Wang, 2016). The bacteriostatic property of TPGs can be exploited for food preservation. Mao Jun‐long TFs treatment reduced the growth of Acinetobacter, Myroides, psychrophilic bacteria, and Flavobacterium on the surface of large yellow croakers and protected the umami taste. Furthermore, the antioxidant properties of TFs also helped delay the spoilage of fat‐rich large yellow croaker (Cui et al., 2020). TBs were shown to have a strong antibacterial effect against *Staphylococcus aureus*, while probiotics such as *Lactobacillus casei*, *Lactobacillus delbrueckii*, and *Enterococcus faecalis* were not affected (Lv et al., 2019). The degree of binding between polysaccharides and polyphenols in TBs determines the bacteriostatic effect; a higher phenol content produces higher bacteriostatic activity.

Mechanistically, TPGs may increase bacterial cell membrane permeability, induce coagulation of bacterial proteins, destroy the bacterial cell membrane structure, reduce adhesion, inhibit bacterial transcription, and/or combine with bacterial DNA; all of these alter the physiological state of bacteria, inhibiting their growth (Chen & Zhen, 2014; Halliwell, 2007).

#### 2.3.4 Antioxidative activity

TPGs can exert antioxidative effects by scavenging free radicals (Kensler et al., 2007), chelating metal ions (Feng et al., 2002), activating the activity of antioxidant enzymes, and regulating related cellular pathways (Luczaj & Skrzydlewska, 2005). As a natural antioxidant, TFs have significantly stronger DPPH free radical scavenging, 2,2'‐azino‐bis(3‐ethylbenzothiazoline‐6‐sulfonate) Diammonium scavenging, and Cu^2+^ reducing activities than Trolox (Wang, 2007). Their antioxidant effects may involve hydrogen atoms and electron transfer. TFs can be converted into stable o‐benzoquinone products. TRs too have free radical scavenging and anti‐oxidation properties similar to catechins and TFs. Luczaj and Skrzydlewska (2005) showed that TRs in black tea can inhibit the generation of free radicals, scavenge free radicals, and chelate transition metal ions. TFs and TRs can affect the activation of NF‐κB or AP‐1 transcription factors. TRs have a lower antioxidant capacity than EGCG and TFs. Wu et al. (2017) showed that TRs not only scavenge free radicals but also have a strong inhibitory effect on O^2−^. Shon et al. (2007)) extracted TRs from Korean microbial fermented tea and found it had a 97% OH· scavenging rate at 80 μg/mL. Yang et al. (2007) found that TRs have a strong ability to scavenge DPPH and OH. TRs can activate the catalase (CAT), which decomposes H_2_O_2_ into H_2_O and O_2_ and inhibit the formation of OH· from H_2_O_2_ and O_2_ under iron ion catalysis.

TRs are also effective antioxidants and metal‐chelating agents. He et al. (2012) found that TRs had a higher hydroxyl radical scavenging rate than ascorbic acid (Vc); the maximum scavenging rate of TRs was 74%. Zhang et al. (2016) found that TRs DPPH free radical scavenging rate decreased with an increase in acidity. Ni et al. (2010) measured the hydroxyl radical (•OH) and superoxide anion radical (•O^2−^) scavenging activity of Pu'er tea extract and TBs. They found that at 20 and 25 mg/mL, respectively, TB and Pu‐erh tea extracts produced the maximum scavenging activity. Also, the scavenging rate of TBs was significantly stronger than that of Pu‐erh tea extract. He et al. (2018) also showed that TBs had high scavenging activity. The antioxidant activity increased in a dose‐dependent manner; TB's clearance rate was 68.6% at 5 g/L.

The above studies somewhat explain the antioxidant mechanism of TPGs, which is mostly related to their structure. However, the underlying chemical mechanisms are not yet fully clear and demand further studies.

## 3 LIMITATIONS AND POTENTIAL APPLICATIONS OF TPGS

**FIGURE 5:**
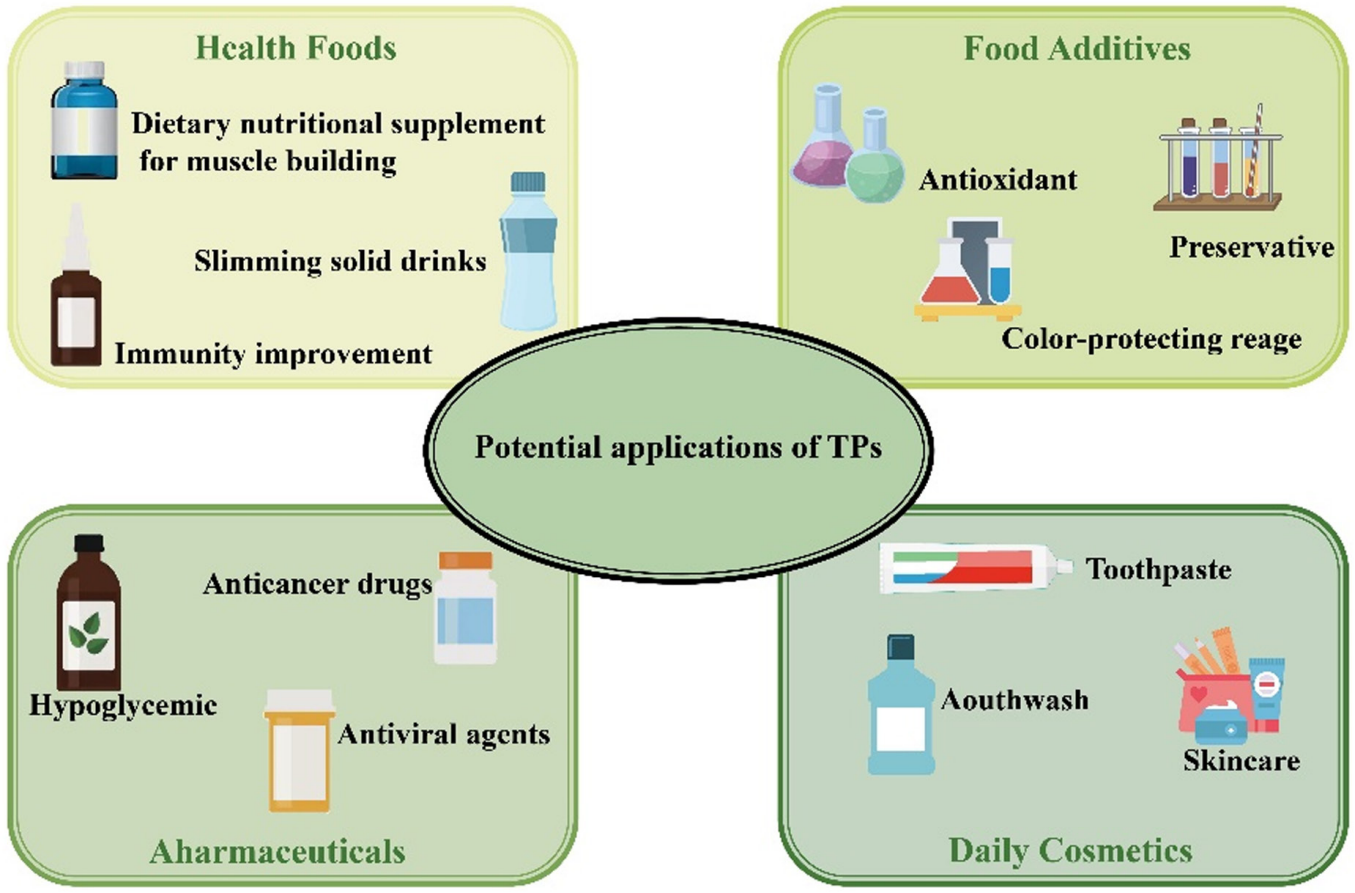
Applications of TPGs.

With a long planting history, China produces more than 300,000 tons of tea annually and has rich tea resources. There is an increase in various metabolic syndromes and chronic diseases caused by unhealthy living habits, such as smoking, alcohol abuse, staying up late, high‐fat diets, and a lack of exercise. Therefore, research and development of healthy foods are strongly encouraged. TPGs are widely used in industries such as food, medicine, health products, and cosmetics (Figure 5).

The health benefits of TPGs are evident from the recent patents. Zhang, Miao, et al. (2022), Zhang, Qu, et al. (2022) obtained the patent “A dietary supplement of theaflavin to strengthen muscles and its preparation method” using TF as a raw material to prepare a dietary supplement that can promote muscle regeneration, inhibit muscle protein degradation and oxidative damage, and improve exercise capacity. Tan et al. (2013) obtained the patent “A health food or drug of theaflavin and its preparation method”. Based on tea seed oil, TFs were formulated into healthy foods for synergistic antioxidation, blood lipid regulation, immunity enhancement, antitumor, and other effects. Sheng Jun et al. (Sheng & Zhao, 2021) obtained the patent “preparation method of theabrownin fermentation broth extract and weight‐reducing solid beverage of theabrownin”, which solves the low purity problem of *Aspergillus niger*‐fermented TBs and optimizes the process of low‐fat solid beverage.

The National Health and Family Planning Commission listed TFs as food additives in 2016. As a green additive, TFs can prevent the discoloration of desserts and cakes, inhibit bacteria, and improve the quality of cakes. Also, they can be used in meat and aquatic products for preservation, color protection, and bacteriostasis. Sun et al. (2017)) showed that TFs can be used as a natural antioxidant to control the melanosis of shrimp. Mao (2021), (Cui et al., 2020) proved that TFs slowed the decomposition of protein and lipid in large yellow croaker, which significantly delayed the quality changes in fish during frozen storage. Therefore, TPGs, in combination with other preservatives or modern biotechnology, can play an important role in food preservation.

In terms of medicine, TFs are used in the Japanese pharmaceutical industry as raw materials for antitoxins to treat intestinal infections, toxic symptoms, etc. TFs can also be used as raw materials for glucagon inhibitors. In recent years, China has also begun to use TFs for medical purposes. Wang et al. (2022) obtained the patent “Applying theaflavins to the drugs of ovarian function protection” to treat chemotherapy‐induced ovarian injury in mammals. With anti‐cancer effects and fewer adverse effects, TFs can prevent chemotherapy‐caused ovarian damage and possibly repair the damaged site. Zhang (2022a, 2022b) obtained the patents “Applying theabrownin to drugs to treat liver cancer” and “Applying theabrownin to anti‐melanoma drugs” Zhang (2022a, 2022b). They proved that TBs promote the apoptosis of hepatocellular carcinoma Huh7 cells by activating the JNK signaling pathway and inhibit the growth of transplanted hepatocellular carcinoma and the proliferation of A375 cells. Therefore, TBs can be used as anti‐hepatoma and anti‐melanoma drugs.

TPGs are widely used in the cosmetic industry. Kong (2022)) showed that TFs containing toothpaste can steadily adjust the group of oral bacteria in saliva and supragingival plaque, improving the core oral microbiome. “An anti‐glycation and whitening theaflavin composition and its application” developed by Ren Xueyin (Ren et al., 2021), et al., used TFs in health care products to improve skin yellowing and pigmentation caused by glycation, oxidation, and melanin. Cai et al. (2022) obtained the patent “A theaflavin composition and its application” showing that TFs can improve the activity of antioxidant enzymes, repair mitochondrial membrane potential, and reduce oxidative damage caused by UVB radiation. TF‐3‐G was shown to effectively inhibit the inflammatory response caused by UVB radiation and reduce the intracellular aggregates in HaCaT cells. It may have broad prospects for photoaging skin care products or pharmaceuticals.

## 4 CONCLUSIONS AND PERSPECTIVES

This paper summarizes selected research on the health benefits of TPGs. Based on the current dietary concept of “homology of medicine and food “, people are more interested in green products. Therefore, the biological activity and health benefits of natural products such as TPGs have turned them into research hotspots. TPGs are widely used in health care, daily chemical products, and other fields.

The difficulties in the research and development of TPGs restrict their development. Except for TFs, the structural details of other TPGs, such as TRS and TBs, are not clear. Therefore, future research should focus on understanding the mechanisms and structural types of TPGs, which may help understand their biological and functional activities. Furthermore, high‐throughput sequencing technology, enzyme technology, and clinical studies can significantly improve the understanding of TPGs by examining details such as metabolism, bioavailability, validity, and biosafety. Also, studies need to solve the difficulties of TPGs' mass production. All of these research activities can boost the development and application of TPG‐based products. There is still a long way to go before realizing the full potential of TPGs. The current trends suggest a promising future for TPGs.

## AUTHOR CONTRIBUTIONS

**Xuan Zhao:** Writing – original draft (equal). **Fei Yan:** Conceptualization (equal); funding acquisition (lead); supervision (equal). **Xin‐Sheng Li:** Conceptualization (equal); supervision (equal). **Dong Qu:** Writing – review and editing (equal). **Yue‐Ling Xu:** Writing – review and editing (equal).

## FUNDING INFORMATION

This research was funded by the Project of Shaanxi Provincial Department of Science and Technology (NO. 2021NY‐044, NO. QBXT‐Z(Z)‐15–2, NO. 2018SZS‐27‐05), and Project of Shaanxi University of Technology (NO. SLGZX2104).

## CONFLICT OF INTEREST STATEMENT

All authors declare no conflict of interest.

## CONSENT TO PARTICIPATE

All the co‐authors are willing to participate in this manuscript.

## CONSENT FOR PUBLICATION

All the authors are willing for the publication of this manuscript.

## Data Availability

The data that support the findings of this study are available from the corresponding author upon request.
